# Antiretroviral therapy (ART) for treating HIV infection in ART-eligible pregnant women

**DOI:** 10.1590/S1516-31802010000400016

**Published:** 2010-07-01

## Abstract

**BACKGROUND:**

This systematic review focuses on antiretroviral therapy (ART) for treating human immunodeficiency virus (HIV) infection in ART-eligible pregnant women. Mother- to-child transmission (MTCT) is the primary means by which children worldwide acquire HIV infection. MTCT occurs during three major timepoints during pregnancy and the postpartum period: in utero, intrapartum, and during breastfeeding. Strategies to reduce MTCT focus on these periods of exposure and include maternal and infant use of ART, caesarean section before onset of labour or rupture of membranes, and complete avoidance of breastfeeding. Where these combined interventions are available, the risk of MTCT is as low as 1-2%. Thus, ART used among mothers who require treatment of HIV for their own health also plays a significant role in decreasing MTCT.

**OBJECTIVE:**

Our objective was to assess the current literature regarding the treatment of HIV infection in pregnant women who are clinically or immunologically eligible for ART. This review includes an evaluation of the optimal time to start therapy in relation to the woman’s laboratory parameters and/or gestational age. It also includes an analysis of which specific antiretroviral medications to start in women who are not yet on ART and which agents to continue in women who are already on ART.

**CRITERIA FOR CONSIDERING STUDIES FOR THIS REVIEW:**

In June 2009, electronic searches were undertaken in these databases: Cochrane’s "CENTRAL," EMBASE, PubMed, LILACS, and Web of Science/Web of Social Science. Hand searches were performed of the reference lists of all pertinent reviews and studies identified. Abstracts from relevant conferences were searched. Experts in the field were contacted to locate additional studies. The search strategy was iterative.

**SELECTION CRITERIA:**

We selected randomized controlled trials and observational studies that evaluated pregnant women with HIV infection who were eligible for ART according to criteria defined by the WHO guideline review committee. Studies were included in the systematic review when a comparison group was clearly defined and where the intervention comprised triple ART. For a study to be considered, each medication in the ART regimen needed to be clearly described.

**DATA COLLECTION AND ANALYSIS:**

Two authors independently assessed the selected studies for relevance and inclusion. Relevant data was then extracted from included studies, and the risk of bias assessed. In each included study, the relative risk (RR) for the intervention versus the comparison group was calculated for each outcome, as appropriate, with 95% confidence intervals (CIs).

**MAIN RESULTS:**

To our knowledge, there are no randomized controlled trials or observational studies that address the optimal time to start antiretroviral drugs in ART-eligible pregnant women in relation to the woman’s laboratory parameters and/or gestational age. The medications to continue in ART-eligible pregnant women who are already receiving ART also have not been evaluated systematically in the current literature. The long-term mortality of HIV-positive pregnant women on ART for their own health, and the long-term virologic or clinical efficacy of ART in treating them, has not been evaluated in randomized clinical trials. In this review, surrogate outcomes for long-term mortality and virologic and clinical efficacy (e.g. MTCT and infant HIV transmission or death) were evaluated to determine the efficacy of specific antiretroviral regimens to start in women who are not yet on ART. In June 2009, electronic searches were undertaken in these databases: Cochrane’s "CENTRAL," EMBASE, PubMed, LILACS, and Web of Science/Web of Social Science. Hand searches were performed of the reference lists of all pertinent reviews and studies identified. Abstracts from relevant conferences were searched. Experts in the field were contacted to locate additional studies. The search strategy was iterative.

**AUTHORS’ CONCLUSIONS:**

In ART-eligible pregnant women with HIV infection, ART is a safe and effective means of providing maternal virologic suppression, decreasing infant mortality, and reducing MTCT. Specifically, AZT/3TC/NVP, AZT/3TC/LPV-r, and AZT/3TC/ABC have been shown to decrease MTCT. More research is needed regarding the use of specific regimens and their maternal and infant side-effect profiles.

**FURTHER INFORMATION:** Centro Cochrane do Brasil Rua Pedro de Toledo, 598 Vila Clementino — São Paulo (SP) — Brasil CEP 04039-001Tel. (+55 11) 5579-0469/5575-2970 http://www.centrocochranedobrasil.org.br/

## COMMENTS

Without any intervention, the risk of mother-to-child transmission (MTCT) of HIV is around 25.5%. MTCT occurring at the time of partum and during labor accounts for 65% of such transmission, while occurrences in uterus account for 35%. Breastfeeding by HIV-positive mothers accounts for around 7 to 22% of HIV transmission to children. Several studies have demonstrated reductions in MTCT of between 0 and 22%, through the following interventions: reduction of the viral load to less than 1000 copies/ml at the end of pregnancy; caesarean section before the onset of labor or rupture of membranes; and prophylactic administration of antiretroviral therapy (ART) for pregnant women, newborns and during breastfeeding, using AZT.

In 1994, the results from Protocol 076 (Pediatric AIDS Clinical Trial Group; PACTG) were published in New Engl J Med. 1994:331(18):1173-1180. This randomized, placebo-controlled, double blind trial demonstrated that for pregnant women with CD4 cell counts of more than 200 cells/mm^3^, AZT administration before labor and during delivery and for newborns during the first six weeks of life reduced MTCT of HIV by 67.5%.

Protease inhibitors were introduced in 1997, in association with other forms of ART for HIV treatment. Through this, it was possible to reduce the viral load to undetectable levels, i.e. below the threshold of laboratory methods at that time. Morbidity and mortality were greatly reduced. Furthermore, the use of ART consisting of a combination of three drugs was capable of achieving important reductions in viral load and MTCT, down to levels close to 0%.

The WHO guidelines and the Brazilian STD/AIDS program consider that patients are eligible to start ART if they present CD4 cell counts lower than 350/mm^3^. In the 1994 study mentioned above, pregnant women were included only if their counts were more than 200 cells/mm^3^, and therefore both eligible women and non-eligible women (CD4 counts of more than 350 cells/mm^3^) were included in the study. Nowadays, all HIV-positive pregnant women should receive ART, whether their CD4 cell count is more than or less than 350 cells/mm^3^, in order to prevent MTCT. Pregnant women who started treatment with counts of more than 350 cells/mm^3^ can stop ART under medical discretion until such time that their cell counts go down to the established threshold, according to the guidelines.

The lack of randomized studies on the issues mentioned in this review does not decrease the importance of testing all pregnant women for HIV. Women who test positive should receive ART or prophylaxis for HIV, in order to avoid MTCT and preserve their health. It seems unnecessary to perform randomized studies to evaluate the optimal time to start ART among eligible pregnant woman, given that there is so much good evidence regarding the benefits of ART starting when the CD4 cell count is below 350 cells/mm^3^, both among pregnant and among non-pregnant women. With regard to the treatment regimens mentioned, it should be borne in mind that nevirapine produces liver toxicity in women with CD4 cell counts of more than 250 cells/mm^3^.

In clinical practice, this review does not modify the established approaches relating to pregnant women, and the studies suggested in the review will only add better knowledge of the evolution of HIV infection in women, which is already the subject of studies among non-pregnant women.

